# Comment on “Calcium Hydroxyapatite and Polymicronutrient Solution on Hand Rejuvenation: A Split‐Hand, Randomized, Double‐Blind Clinical and In Vitro Study”

**DOI:** 10.1111/jocd.71211

**Published:** 2026-10-14

**Authors:** Juyan Song, Haihong Wang, Jie Shen, Honglian Huang

**Affiliations:** ^1^ Department of Dermatology Guiyang First People's Hospital Guiyang China

**Keywords:** calcium hydroxyapatite, equivalence, hand rejuvenation, hyaluronic acid, methodology

To the Editor,

We read with great interest the study by Faria et al. evaluating calcium hydroxyapatite (CaHA) combined with a polymicronutrient (PMN) solution for hand rejuvenation [1]. The authors are to be commended for conducting a randomized, double‐blind, split‐hand clinical trial together with complementary in vitro experiments. Nevertheless, several methodological considerations warrant attention when interpreting the comparative clinical findings and the proposed biological mechanisms.

A principal interpretive issue concerns component‐specific attribution. The PMN formulation contains a mixture of micronutrients together with non‐cross‐linked hyaluronic acid (HA) at a concentration of 10 mg/mL [1]. Non‐cross‐linked HA is itself biologically active and has recognized hydration‐related and skin‐rejuvenating properties [2]. The clinical comparison therefore evaluates CaHA combined with the complete PMN formulation against CaHA diluted with saline; it does not isolate the contribution of the non‐HA constituents from that of HA. Similarly, although the in vitro experiments demonstrate biological activity, they do not distinguish the effects of HA from those of the remaining constituents of the PMN formulation [1]. Accordingly, the incremental clinical or biological effects attributable specifically to the non‐HA constituents cannot be established within the present experimental framework.

A second and particularly important consideration concerns the interpretation of the comparative statistical findings. The absence of a statistically significant between‐treatment difference should not be interpreted as evidence of therapeutic equivalence. The reported analyses were based on conventional null‐hypothesis significance testing rather than a prespecified equivalence or non‐inferiority framework [1, 3]. Although a 1‐point between‐hand difference on the Hand Grading Scale was identified as clinically relevant, confidence intervals for the between‐treatment effect were not reported in relation to this threshold [1]. Given the small sample size (*n* = 22) and the absence of a reported power calculation, a nonsignificant between‐treatment result may also reflect limited precision or statistical power. The findings therefore indicate that a difference was not detected; they do not establish therapeutic equivalence or non‐inferiority between the two treatment regimens [3].

A further important consideration concerns the mechanistic interpretation of the injection technique and the in vitro observations. In the clinical study, the formulations were administered using a 22G × 50 mm cannula into the superficial layer of the dorsal hand, specifically the potential space between the dermis and the superficial fascia, with advancement in the subdermal plane [1]. The authors appropriately explain that this avascular and tendon‐free plane was selected to minimize procedural risk and facilitate even product‐distribution. However, the formulations were deposited in the subdermal plane rather than directly within the dermis, whereas the in vitro experiments involved direct exposure of cultured dermal fibroblasts to the test formulations. No histological or tissue‐distribution assessment was performed to demonstrate migration or diffusion of the formulation into the overlying dermis. Consequently, although effects on the dermis remain biologically plausible, the clinical observations cannot by themselves establish that the fibroblast responses observed in vitro also occurred in vivo.

The biological interpretation of the clinical findings should therefore be further moderated. Skin hydration was objectively assessed by corneometry, and dermal and hypodermal thickness were measured by ultrasound; however, these measurements do not directly demonstrate collagen synthesis, fibroblast activation in vivo, extracellular‐matrix remodeling, or tissue regeneration. No skin biopsy, histological assessment, quantitative collagen analysis, immunohistochemical evaluation, or in vivo gene‐expression analysis was performed. In particular, an increase in ultrasound‐measured dermal or hypodermal thickness cannot distinguish newly formed extracellular matrix from retained product, volumization, fluid‐related changes, or mechanical tissue expansion. Similarly, qualitative ultrasound findings described as being “consistent with collagen remodeling and improved tissue organization” should be interpreted cautiously, because ultrasound morphology alone does not establish collagen remodeling. Accordingly, references to collagen remodeling, dermal regeneration, or enhanced regenerative activity should be framed as mechanistic interpretations supported by prior literature and the in vitro findings, rather than as biological effects directly demonstrated by the clinical component of the present study.

Finally, the small, predominantly female, single‐center sample and the 90‐day follow‐up period limit the generalizability of the findings and conclusions regarding longer‐term comparative outcomes [1]. The clinical study included only 22 participants, 21 of whom were women. Longer follow‐up is particularly important when delayed or cumulative tissue responses are being proposed. These limitations do not detract from the reported improvements in HGS, corneometry‐derived hydration, ultrasound‐measured dermal/hypodermal thickness, investigator‐assessed aesthetic appearance, and patient‐reported satisfaction with either treatment regimen, but they support a more circumscribed interpretation of component‐specific mechanisms and comparative treatment effects.

Future studies incorporating a comparator consisting of CaHA combined with matched non‐cross‐linked HA would help distinguish the contribution of the remaining PMN constituents. Prespecified equivalence or non‐inferiority analyses, together with confidence intervals interpreted against a clinically justified margin, would permit more robust conclusions regarding comparative efficacy. Product‐distribution studies and direct tissue‐level assessments would also help clarify whether the observed changes reflect volumization, hydration‐related effects, dermal remodeling, or a combination of these mechanisms.

We commend Faria et al. for contributing valuable data to the field of hand rejuvenation and look forward to further investigations addressing these questions.

## Author Contributions

Juyan Song and Haihong Wang conceptualized and drafted the letter. All authors critically revised the manuscript and approved the final version.

## Funding

The authors have nothing to report.

## Ethics Statement

The authors have nothing to report.

## Conflicts of Interest

The authors declare no conflicts of interest.

## Data Availability

Data sharing not applicable to this article as no datasets were generated or analysed during the current study.
